# The hemodynamic impact of congenital heart diseases during fetal-to-neonatal transition: an in-silico investigation

**DOI:** 10.1038/s41390-025-04565-1

**Published:** 2026-01-30

**Authors:** Bettine G. van Willigen, Babette C. Krabben, M. Beatrijs van der Hout-van der Jagt, Wouter Huberts, Frans N. van de Vosse

**Affiliations:** 1 Cardiovascular Biomechanics, Biomedical Engineering, Eindhoven University of Technology, Eindhoven, The Netherlands; 2 Obstetrics and Gynaecology, Máxima Medical Centre, Veldhoven, The Netherlands; 3 Signal Processing Systems, Electrical Engineering, Eindhoven University of Technology, Eindhoven, The Netherlands

## Abstract

**Background:**

Congenital heart disease (CHD) is a significant congenital anomaly, with ventricular septal defect (VSD) and transposition of the great arteries (TGA) being commonly encountered. Despite advancements in prenatal diagnosis and postnatal care, predicting neonatal outcomes remains difficult due to fetal compensatory mechanisms. This study utilizes and adapts existing mathematical models, without patient-specific parameters, to simulate the hemodynamic effects of VSD and TGA during the fetal-to-neonatal transition, validated against literature data.

**Method:**

The model uses a closed-loop 0D-1D cardiovascular system and an oxygen-carbon dioxide exchange model to retrieve clinically relevant information.

**Results:**

Results show realistic replication of flow, pressure profiles, oxygen saturation, and carbon dioxide levels for both normal and CHD-compromised fetuses.

**Conclusion:**

The mathematical model offers valuable insights into hemodynamic alterations during the fetal-to-neonatal transition, also under two pathophysiological conditions. It provides a non-invasive means of predicting clinically relevant parameters, which is essential for postnatal treatment planning in the prenatal period, especially given the potential need for immediate postnatal hemodynamic management. Overall, this model presents a promising tool for predicting the impact of CHD postnatally based on prenatal information and for guiding timely and effective treatment strategies. As such, the model may be considered as a potential basis for other applications in perinatal hemodynamics including fetal-to-neonatal transition.

**Impact:**

- This study introduces a mathematical model that simulates the hemodynamic impact of congenital heart disease (CHD) during the fetal-to-neonatal transition, focusing on ventricular septal defect (VSD) and transposition of the great arteries (TGA). By integrating a one-fiber heart model and oxygen and carbon dioxide dynamics, the model gives a prediction of critical clinical parameters like flow, pressure, and oxygen saturation. This mathematical model has a potential to serve as non-invasive tool, providing valuable insights for prenatal planning and postnatal management, helping guide timely interventions for newborns affected by CHD.

## Introduction

### Clinical background

Congenital heart disease (CHD), with a prevalence rate of 170.6 per 100,000 in 2017,^1^ is accounting for approximately one-third of all diagnosed congenital conditions worldwide.^2,3^ CHD is defined as structural abnormalities within the heart and/or great vessels present at birth.^4^ Among the CHDs, ventricular septal defect (VSD) is the most common with an incidence of approximately 3–4 per 1000 live births.^3–5^ Meanwhile, transposition of the great arteries (TGA) is one of the most frequently diagnosed cyanotic cardiac defects, with an incidence of 2–5 per 10,000 live births.^4,6,7^

VSD is characterized by a hole in the ventricular septum, allowing inter-ventricular shunting. The prognosis of neonates with VSD depends on factors, such as the location and size of the defect as well as its isolation or association with other congenital anomalies.^3,8^ Clinical presentations of VSD may include failure to thrive and respiratory distress, with severe cardiac failure potentially emerging within 4–6 weeks after birth.^9^ In patients with TGA, the main pulmonary artery originates from the left ventricle, while the ascending aorta originates from the right ventricle. Newborns with TGA are at high risk of hemodynamic decompensation and can become rapidly cyanotic after birth.^10^

Hence, early diagnosis of CHD is crucial and assessment of prenatal severity of abnormal cardiac physiology allows prediction of the risk of postnatal hemodynamic instability.^10^ Despite advancements in prenatal diagnostics,^11^ congenital diseases remain underdiagnosed, with a detection rate of less than 50%.^12,13^ In addition, when CHD is detected prenatally, predicting the impact of CHD on neonatal outcome based on this assessment, remains challenging as fetal shunts often compensate for the CHDs.^14^ Only after birth, CHDs affect the well-being of the baby, which complicates the prenatal prognosis of CHDs.

### Fetal-to-neonatal transition

The fetal cardiovascular system includes three unique shunts: the foramen ovale, the ductus arteriosus, and the ductus venosus. These shunts facilitate fetal gas exchange, nutrient supply, and waste disposal via the placenta. The placenta oxygenates oxygen-poor blood from the umbilical arteries, resulting in oxygen-rich blood within the umbilical vein.^15^ Originating from the umbilical vein, the ductus venosus transports the highly oxygenated and nutrient-rich blood from the placenta directly to the heart.^16^ Subsequently, this blood is shunted from the inferior vena cava to left atrium via the foramen ovale, facilitating inter-cardiac mixing oxygen-rich and oxygen-poor blood. The ductus arteriosus shunts blood between the main pulmonary artery and the aortic isthmus, directing the blood from the main pulmonary artery to the systemic circulation. Hence, the oxygen-rich blood from the ascending aorta supplies the upper body and brain, while the ductus arteriosus supplies the rest of the body, including the placenta. Thus, these unique shunts ensure intercirculatory mixing of oxygen-rich and oxygen-poor blood and a parallel circulation.^8^

During the transition from fetus to neonate, the closure of these unique fetal shunts and placental circulation occurs either immediately upon umbilical cord clamping (placental circulation), gradually due to vasoconstriction (ductus venosus, ductus arteriosus), or due to change in the pressure gradient (foramen ovale). Umbilical cord clamping, thus the removal of the placental circulation, leads to an increase in systemic vascular resistance and closure of the ductus venosus.^17^ The initial breaths of the newborn cause lung expansion, vasodilate pulmonary circulation, and removal of fluid in the lungs affecting the alveolar partial oxygen pressure. The combination of the enhanced lung surface area and reduced pulmonary vascular resistance amplifies pulmonary blood flow, resulting in a redistribution of the combined cardiac output.^8^ This redistribution alters atrial pressure gradients, which closes the foramen ovale. Additionally, as the partial oxygen pressure of the blood increases, the production of prostaglandin E2 diminishes, stimulating ductus arteriosus constriction and closure.^18^ When these unique shunts are closed, the cardiovascular transition from fetus to neonate is completed, resulting in separation of the left and right side of the heart and an in series circulation.

Clinical reference data regarding this transition in the context of CHDs is sparse due to the invasive nature of such measurements. However, the acquisition of such data is crucial for defining a postnatal treatment plan prenatally, especially considering the potential necessity for immediate postnatal hemodynamic management. To overcome these challenges, mathematical models can be established to predict clinically relevant parameters non-invasively. Such models offer insights into hemodynamic alterations during the fetal-to-neonatal transition and various pathophysiological scenarios.

### Existing mathematical models

Previous in silico studies have been performed to investigate the influence of CHDs on the cardiovascular circulation. For instance, Jalali et al.^19^ developed a fetal mathematical model focusing on hypoplastic left heart syndrome with an atrial septal defect, while Zhang et al.^20^ simulated a fetus with TGA. Furthermore, several models have been established for simulating the fetal-to-neonatal transition under healthy conditions.^21–23^ However, as far as could be derived from literature, the hemodynamics during fetal-to-neonatal transition in the presence of CHDs have not yet been reported.

Moreover, the majority of these models use the time-varying elastance model to simulate cardiac adaptations, such as alterations in material properties (e.g., tissue stiffness and contractility) and volume during the transition or in the presence of CHDs. The time-varying elastance model represents both material and geometrical adaptations using a single parameter, elastance. While this approach yields realistic outcomes by adapting the elastance parameter to the specific scenario,^19–22,24^ providing a complete representation of cardiac function across multiple (patho)physiological scenarios is challenging due to the inability to separate material and geometrical changes. In contrast, the one-fiber model^23,25,26^ simulates the relationship between chamber pressure, cavity and wall volume, and myocardial stress, thereby offering an approach to describe cardiac function by separating material and geometrical properties. Thus, it is hypothesized to capture cardiac function in more detail and more realistically, particularly in the context of pathophysiological scenarios. Therefore, the one-fiber model potentially offers insights into the hemodynamical interplay between CHDs and fetal-to-neonatal transition.

### Aim

The aim of this study is to assess the ability of a closed-loop 0D-1D fetal cardiovascular model, without patient-specific parameters, to simulate the hemodynamic impact of VSD and TGA during fetal-to-neonatal transition, validated against literature data. This approach gains understanding of the hemodynamics of CHDs during fetal-to-neonatal transition, serving as a first step toward detecting CHDs and improving treatment planning. This study is part of a larger fetal circulatory framework that eventually aims to provide a digital twin of the fetal circulation.^27^ To achieve this objective, the study integrates the one-fiber model into a simplified closed-loop 0D-1D fetal cardiovascular system, necessitating the incorporation of wave propagation dynamics within the vasculature. In addition, an oxygen and carbon dioxide model have been added to retrieve clinically relevant information.

## Methods

**Fig. 1:**
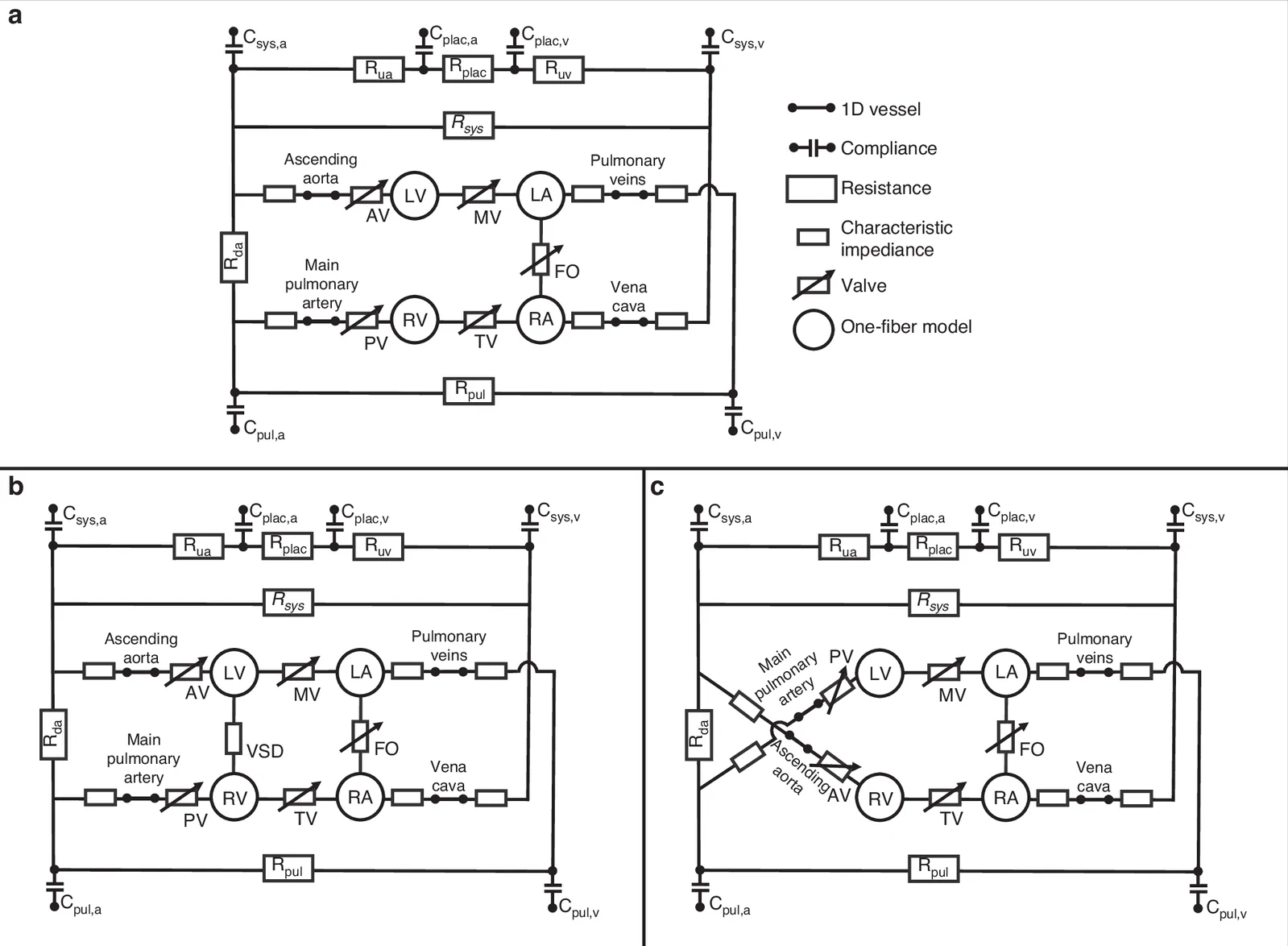
Schematic drawings of the fetal blood circulation model. Panels illustrate the model (**a**) without CHD, **b** with VSD, and **c** with TGA. Each model consists of one-fiber models representing cardiac chambers, large vessels described with a 1D model, and lumped parameter elements representing the cardiovascular circulation. LA: left atrium, LV: left ventricle, RA: right atrium, RV: ventricle, ${{{\rm{C}}}}_{{{\rm{pul}}},{{\rm{a}}}}$: pulmonary arterial compliance, ${{{\rm{R}}}}_{{{\rm{pul}}}}$: pulmonary resistance, ${{{\rm{C}}}}_{{{\rm{pul}}},{{\rm{v}}}}$: pulmonary venous compliance, ${{{\rm{C}}}}_{{{\rm{sys}}},{{\rm{a}}}}$: systemic arterial compliance, ${{{\rm{R}}}}_{{{\rm{sys}}}}$: systemic resistance, ${{{\rm{C}}}}_{{{\rm{sys}}},{{\rm{v}}}}$: systemic venous compliance, ${{{\rm{R}}}}_{{{\rm{ua}}}}$: umbilical arterial resistance, ${{{\rm{C}}}}_{{{\rm{plac}}},{{\rm{a}}}}$: placental arterial compliance, ${{{\rm{R}}}}_{{{\rm{plac}}}}$: placental resistance, ${{{\rm{C}}}}_{{{\rm{plac}}},{{\rm{v}}}}$: placental venous compliance, ${{{\rm{R}}}}_{{{\rm{uv}}}}$: umbilical venous resistance.

**Fig. 2:**
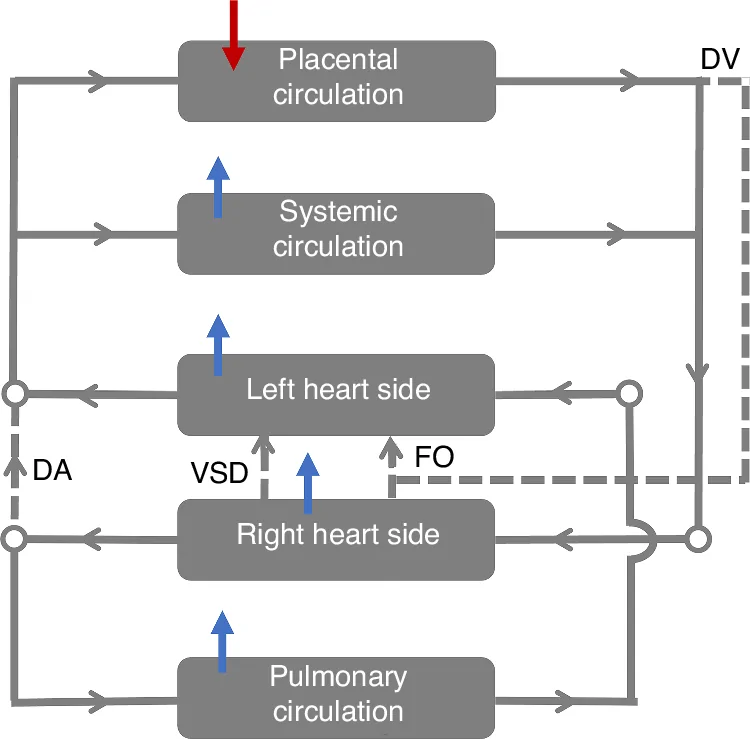
A schematic drawing of blood gas model. The blood gas model consists of compartments matching the fetal blood circulation model. The red and blue arrows represent oxygen inflow and consumption, respectively.

This study uses the mathematical foundation of previously developed models of fetal cardiovascular physiology: the blood circulation model^28^ and the blood gas model.^29^ The former integrates a cardiac function model, valve model, 1D representation of the larger vessels, and 0D elements for microcirculations (Fig. 1a). The fetal blood circulation model is translated into gas exchange compartments and contains models for oxygen and carbon dioxide transport (Fig. 2). The inflow and volume of these compartments are input parameters derived from the blood circulatory model output. The following sections delve into the mathematical framework of these models in their fetal state, followed by an explanation of controlled parameters adjustments required for the fetal-to-neonatal transition. Additionally, the methodology for incorporating VSD (Fig. 1b) and TGA (Fig. 1c) is given.

### Blood circulation model

#### Cardiac function

In this model, cardiac function is described by the one-fiber model, which simulates the relationship between cardiac pump function and myofiber mechanics. Initially proposed by Arts et al.,^25^ the one-fiber model assumes the cardiac cavity to be wrapped by a single long fiber. The pressure $({p}_{{cav}})$ within each cardiac chamber associates with myocardial stress (${\sigma }_{m}$), cavity volume (${V}_{{cav}}$), and wall volume (${V}_{{wall}}$), according to^26,30^:

1 $${p}_{{cav}}\left(t\right)=\frac{1}{3}{\sigma }_{m}\left(t\right){ln}\left(1+\left(\frac{{V}_{{wall}}}{{V}_{{cav}}\left(t\right)}\right)\right)$$

Here, the myocardial stress ${\sigma }_{m}\left(t\right)$ represents the driving force based on time dependent active behavior of myocardial fibers. Previous work^28^ gives a detailed description of the one-fiber model and the present study utilizes the same model parameters.

#### Valves

As the cardiac valves are conceptualized as diodes, the valves have two states, completely open or closed. The valve state depends on the difference between the proximal pressure ${p}_{p}$ and distal ${p}_{d}$ pressure across the valve, as given by:

2 $${q}_{v,i}=\left\{\begin{array}{cc}\frac{{p}_{p,i}-{p}_{d,i}}{{R}_{v,i}}&{p}_{p,i} > {p}_{d,i}\hfill \\ 0\hfill &{p}_{p,i}\le {p}_{d,i}\end{array}\right.$$

Here, the terms proximal and distal in relation to the valves refer to the positions upstream and downstream of the mean flow through the valve, respectively. The ${R}_{v,i}$ represents the resistance of valve i, where i ∈ (MV, TV, AV, PV, FO) with MV representing the mitral valve, TV the tricuspid valve, AV the aortic valve, PV the pulmonary valve, and FO the foramen ovale. The resistances of the semilunar valves are based on the characteristic impedance of the corresponding 1D vessel. The parameters of the other valves are presented in Table 1.

**Table 1:** Valve model parameters for a full-term fetus without CHD, with VSD, and with TGA.

**Parameter**	**Abbreviation**	**Control**	**VSD**	**TGA**	**Unit**	**Ref**
Resistance mitral valve	${R}_{{mv}}$	0.06		0.124	mmHg s/mL	
Resistance tricuspid valve	${R}_{{tv}}$	0.06		0.032	mmHg s/mL	
Resistance foramen ovale	${R}_{{fo}}$		0.1		mmHg s/mL	^22^

#### Cardiac in- and outflow vessels

The modeling approach for the in- and outflow cardiac vessels uses 1D wave propagation, facilitating the integration of the one-fiber model into the broader fetal circulatory framework in the future.^27^ As outlined in,^28^ mathematical modeling of large vessels described by 1D models relies on mass and momentum balance equations governing blood pressure (p) and flow (q), under the assumptions of Newtonian incompressible fluid, symmetric tube geometry, and neglecting leakage (such as small side branches) and body forces (e.g. gravity forces). To express wall shear stress and steady inertia forces in terms of p and q, an approximate velocity profile proposed by Bessems et al.^31^ is utilized. Additionally, a constitutive relation for arteries and veins is defined to characterize the pressure-area relationship using area compliance.^32^ Arteries are modeled as thin-walled linear elastic tubes as the wall thickness-to-diameter ratio is smaller than 0.1. The wall behavior of thin-walled venous vessels is described by a power law accounting for vessel collapse.^24^ Thick-walled linear elastic tube assumptions^33,34^ are applied for the umbilical arteries and vein due to their larger wall thickness-to-diameter ratio of 0.31. For comprehensive details on 1D mass and momentum balance, the approximate velocity profile, and constitutive relations, see van Willigen et al.^28^ and for the model parameters, see Table 2.

**Table 2:** Geometrical properties of the larger vessels.

Parameter	Symbol	AscAo	MPA	PVen	VC	Unit	Ref
Vessel length	L	1.00	1.72	1.50	0.90	cm	^20^
Vessel radius at reference pressure	${r}_{0}$	4.35	5.00	2.33	6.65	mm	^20^
Young’s Modulus	E	63.5	63.5	95.4	51.6	kPa	^20^
Wall thickness	${h}_{0}$	0.68	0.69	0.15	0.25	mm	^20^

*AscAo* ascending aorta, *MPA* mean pulmonary artery, *PVen* pulmonary veins, *VC* vena cava.

#### Lumped cardiovascular system

As this study primarily focuses on cardiac function, the cardiovascular system is simplified by lumping the entire system except for the cardiac in- and outflow tracts. Figure 1 provides a schematic representation of this model. The systemic circulation is characterized by a resistance (${R}_{{sys}}$), arterial compliance (${C}_{{sys},a}$), and venous compliance (${C}_{{sys},v}$). Similarly, the pulmonary circulation entails a resistance (${R}_{{pul}}$), arterial compliance (${C}_{{pul},a}$), and venous compliance (${C}_{{pul},v}$). The placental circulation involves resistances for the umbilical arteries, umbilical vein, and placental microcirculation (${R}_{{ua}}$, ${R}_{{uv}}$, and ${R}_{{plac}}$), as well as an arterial compliance (${C}_{{plac},a}$) and venous compliance (${C}_{{plac},v}$). External pressure ${p}_{{ext}}$ = 0 mmHg acts on all compliances. Additionally, the systemic and pulmonary circulations are linked by a resistance describing the ductus arteriosus (${R}_{{da}}$). The ductus venosus is lumped with the umbilical vein. The majority of the parameter values are based on previous studies^21,22,24^ (Table 3).

**Table 3:** Lumped parameter model parameters

	Parameter	Abbreviation	Control	VSD	TGA	Ref
**Resistance[mmHg s/mL]**	Pulmonary vascular	${R}_{{pul}}$	8.00		4.56	^21^
	Systemic vascular	${R}_{{sys}}$	2.30			^24^
	Placental	${R}_{{plac}}$	0.10			^21^
	Umbilical artery	${R}_{{ua}}$	3.08			^21^
	Umbilical vein	${R}_{{uv}}$	0.56			^21^
	Ductus arteriosus	${R}_{{da}}$	0.12		0.45	^6,21^
	VSD	${R}_{{vsd}}$		0.10		
**Compliance [mL/mmHg]**	Pulmonary arterial	${C}_{{pul},a}$	0.16			^21^
	Pulmonary venous	${C}_{{pul},v}$	0.49			^21^
	Systemic arterial	${C}_{{sys},a}$	0.29			^21^
	Systemic venous	${C}_{{sys},v}$	10.5			
	Placental arterial	${C}_{{plac},a}$	0.50			^21,22^
	Placental venous	${C}_{{plac},v}$	1.00			^21,22^

To prevent non-physiological wave reflections of high frequencies between the 0D and 1D elements, characteristic impedances (Z) are introduced to connect them:

3 $${Z}_{j}=\sqrt{\frac{\rho }{{A}_{\left({ref},j\right)}{C}_{j}^{1D}}}$$

where ${C}_{j}^{1D}$ and ${A}_{{ref},j}$ are the compliance and the cross-sectional area at reference transmural pressure, respectively, of the connected 1D vessel j.

### Blood gas model

#### Oxygen transport

The rate of change in oxygen within a compartment is governed by oxygen transport through three mechanisms: convection ${q}_{c,{O}_{2}}$, diffusion ${q}_{d,{O}_{2}}$, and metabolic uptake ${q}_{m,{O}_{2}}$, as expressed by:

4 $$\frac{d\left({c}_{{O}_{2}}^{i}{V}^{i}\right)}{{dt}}={q}_{c,{O}_{2}}^{i}+{q}_{d,{O}_{2}}^{i}-{q}_{m,{O}_{2}}^{i}$$

where ${c}_{{O}_{2}}^{i}$ and ${V}^{i}$ represent the volume fractional oxygen concentration and the volume of compartment i, respectively. The oxygen flux by convection considers preferential flow via the ductus venosus.^22^ Oxygen diffusion occurs either in the placenta or lungs, depending on the oxygen diffuse conductance and the partial oxygen pressure difference over the diffusion area.^22,35^ For model parameters, the reader is referred to previous studies.^29^ Metabolic rate is based on the study of Rudolph^14^ and can be found in Table 4.

**Table 4:** Overview of the percentage metabolic rate and threshold ${p}_{{O}_{2,{th}}}$ per blood gas model compartment for the simplified fetal cardiovascular system geometry

Compartment	Metabolic rate [%]	${p}_{{O}_{2,{th}}}$[mmHg]	Ref
Left heart side	6.0	10.0	^14,36^
Right heart side	6.0	10.0	^14,36^
Placental circulation	–	–	–
Systemic circulation	80.0	10.0	–
Pulmonary circulation	8.0	10.0	^14,36^

#### Carbon dioxide transport

Similar to oxygen transport, the rate of change in carbon dioxide within a compartment is influenced by carbon dioxide transport through convection ${q}_{c,{{CO}}_{2}}$, diffusion ${q}_{d,{{CO}}_{2}}$, and excretion ${q}_{e,{{CO}}_{2}}$, given by:

5 $$\frac{d\left({c}_{C{O}_{2}}^{i}{V}^{i}\right)}{{dt}}={q}_{c,{{CO}}_{2}}^{i}-{q}_{d,{{CO}}_{2}}^{i}+{q}_{e,{{CO}}_{2}}^{i}$$

where ${c}_{C{O}_{2}}^{i}$ represents the carbon dioxide concentration fraction of compartment i. Carbon dioxide diffusion takes place either in the placenta or lungs, leaving the fetus to the mother. The flux depends on the carbon dioxide diffuse conductance and the partial carbon dioxide pressure difference over the diffuse area. The carbon dioxide excretion depends on the metabolic rate of the compartment and respiratory quotient. Subsequently, the ${p}_{C{O}_{2}}$ is estimated by a relation on carbon dioxide concentration using Henry’s law.^36^ Model parameters can be found in van Willigen et al.^29^

### Fetal-to-neonatal transition

During birth, the fetal circulation undergoes adaptations, transitioning into neonatal physiology. This is simulated by increasing umbilical arterial (${R}_{{ua}}$) and umbilical venous resistance (${R}_{{uv}}$) to mimic cord clamping, followed by elevation of ${R}_{{da}}.$^23,37,38^ Closure of the foramen ovale is a model result because of its representation as a diode-like valve (see Eq. 2).

Lung aeration is simulated by a linear increase of the conductance of oxygen and carbon dioxide of the lungs (${D}_{{pul},\,{O}_{2}}$ and ${D}_{{pul},{C}{O}_{2}}$, respectively).^39^ In addition, lung aeration causes a decrease in pulmonary vascular resistance (${R}_{{pul}}$)^23^ and pulmonary compliance parameters (${C}_{{pul},a}$ and ${C}_{{pul},v}$) experience an exponential increase.^40,41^

The removal of the placenta significantly contributes to the rise in systemic vascular resistance ${R}_{{sys}}.$^21,42^ A high concentration of catecholamines decreases the systemic compliances (${C}_{{sys},a}$ and ${C}_{{sys},v}$). For an elaborate description and mathematical notation, see Supplementary Material.

### Congenital heart diseases

#### Ventricular septal defect

The intra-ventricular opening is approximated by a resistance (Fig. 1b), simulating a large VSD with a diameter of 6.0 mm.^3^ Since the foramen ovale has a similar diameter,^43^ the VSD resistance ${R}_{{vsd}}$ is set to the same value as the foramen ovale resistance ${R}_{{fo}}$ (see Table 3).

#### Transposition of the great arteries

In fetuses with TGA, the ascending aorta originates from the right ventricle, while the main pulmonary artery is connected to the left ventricle (Fig. 1c). Additionally, the morphometry of the intracardiac valves is adjusted. The increased volume in the right atrium results in an increase of tricuspid valve area,^7^ while the mitral valve area is decreased^7^ (Table 1). Additionally, the ductus arteriosus diameter is smaller in fetuses with TGA compared to healthy fetuses, potentially due to the increased oxygen content in the pulmonary artery.^6,44^ Therefore, the fetal ${R}_{{da}}$ is adjusted to match a radius ${r}_{{da},0}$ of 1.8 mm^6^ (Table 3). Furthermore, the high oxygen-saturated left ventricular blood flowing through the pulmonary artery results in a lower pulmonary vascular resistance for fetuses with TGA.^44–46^ As the pulmonary blood flow is increased by 57%, the fetal pulmonary vascular resistance ${R}_{{pul},0}$ is decreased by 43% for the TGA case. The scaling parameters ${m}_{{pul},1}$ and ${m}_{{pul},3}$ are adapted, such that neonatal endpoint is equal to the control case (Table S.1).

### Numerical implementation

The fetal simulation initialization begins by setting a mean circulatory filling pressure (${p}_{{mcfp}}$) to 8 mmHg, estimated on the unloaded volume and compliance of the circulation.^47^ Subsequently, solving the fetal blood circulation involves an algorithm, consisting of a cardiac cycle and hemodynamic convergence loop. Convergence is achieved when the normalized maximal root mean squared error of pressures and flows between two consecutive cardiac cycles falls below a predefined tolerance (${\varepsilon }_{{tol}}$). Upon hemodynamic convergence, the temporal mean flow and volume of the fetal blood circulation solution serve as the input parameters for the blood gas model, initiating a convergence loop until it achieves convergence. After achieving convergence of the solution of the blood gas model, the fetal-to-neonatal transition begins. Both the blood circulation model and blood gas model are solved every 25 heart cycles (10.7 s) during this transition. For an elaborate explanation of the numerical implementation the reader is referred to.^28,29^ The source code is written in Python and is available upon request. Model-wide simulation parameters can be found in van Willigen et al.^28^.

### Simulations and analysis

In the simulations and analysis phase, the distribution of cardiac output during the fetal-to-neonatal transition is analyzed. This analysis spans multiple time points, including just before birth and 30 s, 10 min, 1 h, 12 h, and 24 h after birth. For each time point, the cardiac output distribution is evaluated for three different scenarios: the control, VSD, and TGA case. Additionally, various parameters are examined for the same time points, including flow and pressure profiles in larger vessels and shunts as well as oxygen saturation (${s}_{{O}_{2}}$) and partial carbon dioxide pressure (${p}_{C{O}_{2}}$) in both left and right ventricular outflow tracts. This analysis provides insights into the hemodynamic changes occurring during the transition from fetus to neonate, and how these changes are influenced by congenital heart defects. No human subjects were involved in the study.

## Results

### Full-term fetus

Table 5 presents the simulated cardiac output distribution of a full-term fetus under various conditions: without CHD (control), with VSD, and with TGA. For the control case, the simulated flow through the shunts and to the sub-circulations is validated against flow measurements conducted with phase-contrast MRI,^48^ demonstrating agreement within a standard deviation. Notably, healthy fetal circulation has typically a dominant right heart (right cardiac output, $${{q}}_{{rco}} > $$ left cardiac output$$,\,{q}_{{lco}}$$), consistent with both simulations and literature data.

**Table 5:** The simulated cardiac distribution of a full-fetus without CHD (control), with VSD, and with TGA compared with literature data in mL/min and percentage of combined cardiac output (%${q}_{{cco}}$)^6,49^

	Control		VSD	TGA	
	Simulation	Prsa et al.^49^	Simulation	Simulation	Lachaud et al.^6^
${q}_{{cco}}$ mL/min (%${q}_{{cco}}$)	1495 (100%)	1702 ± 209	1499 (100%)	1550 (100%)	1279 ± 445
${q}_{{lco}}$ mL/min (%${q}_{{cco}}$)	671 (45%)	699 ± 128 (41 ± 6%)	749 (50%)	586 (38%)	603 ± 275 (47 ± 10%)
${q}_{{rco}}$ mL/min (%${q}_{{cco}}$)	824 (55%)	955 ± 168 (56 ± 6%)	750 (50%)	964 (62%)	683 ± 249 (53 ± 10%)
${q}_{{sys}}$ mL/min (%${q}_{{cco}}$)	774 (52%)		777 (52%)	728 (47%)	828 ± 277
${q}_{{pul}}$ mL/min (%${q}_{{cco}}$)	245 (16%)	271 ± 157 (16 ± 9%)	245 (16%)	374 (24%)	416 ± 238
${q}_{{da}}$ mL/min (%${q}_{{cco}}$)	579 (39%)	684 ± 142 (40 ± 8%)	505 (34%)	212 (14%)	155 ± 117
${q}_{{fo}}$ mL/min (%${q}_{{cco}}$)	426 (28%)	494 ± 179 (29 ± 11%)	403 (27%)	212 (14%)	
${q}_{{uv}}$ mL/min (%${q}_{{cco}}$)	476 (32%)	490 ± 131 (29 ± 9%)	477 (32%)	448 (29%)	
${q}_{{vsd}}$ mL/min (%${q}_{{cco}}$)			101 (7%)		

The data of Lachaud et al. *(6)* represents a fetal age of 35-38 weeks. ${q}_{{cco}}$: combined cardiac output flow, ${q}_{{lco}}$: left cardiac output flow, ${q}_{{rco}}$: right cardiac output flow, ${q}_{{sys}}$: systemic circulation flow, ${q}_{{pul}}$: pulmonary circulation flow, ${q}_{{da}}$: ductus arteriosus flow, ${q}_{{fo}}$: foramen ovale flow, ${q}_{{uv}}$: umbilical vein flow, and ${q}_{{vsd}}$: flow through the VSD.

In the presence of a VSD, the fetal circulation experiences both inter-atrial and inter-ventricular shunting. With a VSD diameter of 6 mm, a right-to-left shunting of 6.8% of combined cardiac output (${q}_{{cco}}$) occurs, reducing flow through the foramen ovale (${q}_{{fo}}$) and ductus arteriosus (${q}_{{da}}$) compared to the control case (${q}_{{fo}}$: 26.9 versus 28.5% of ${q}_{{cco}}$. ${q}_{{da}}$: 33.7 versus 38.7% of ${q}_{{cco}}$). Furthermore, this shunt results in equal dominance of the cardiac sides (${q}_{{rco}}={q}_{{lco}}$). Despite these changes, the total right-to-left shunting remains comparable between the VSD and control cases, resulting in similar pulmonary (${q}_{{pul}}$), systemic (${q}_{{sys}}$), and placental (${q}_{{uv}}$) perfusion, as well as combined cardiac output (${q}_{{cco}}$: 1499 versus 1494 mL/min).

In the TGA case, the ${q}_{{cco}}$ is slightly higher than in the control case (1550 versus 1494 mL/min) due to a 43% decrease in ${R}_{{pul}}$ relative to the control case. This reduction leads to increased ${q}_{{pul}}$ compared to the control case (374 mL/min and 24.1% of ${q}_{{cco}}$ vs. 245 mL/min and 16.4% of ${q}_{{cco}}$, respectively). Conversely, ${q}_{{da}}$ and ${q}_{{fo}}$, both 212 mL/min (13.7% of ${q}_{{cco}}$), are lower compared to the control case (579 and 426 mL/min, respectively). These results closely align with measurements reported by Lachaud et al.^6^ with deviations within a standard deviation, except for the absolute ${q}_{{rco}}$ value only (Table 5). However, note that their measurements correspond to a fetus between 35 to 38 weeks whereas the simulations represent a 40-week-old fetus.

Table 6 presents ${s}_{{O}_{2}}$ and ${p}_{C{O}_{2}}$ of the outflow tracts from both the left and right heart side as well as the umbilical vein. The model results reveal minimal differences with and without CHD in ${s}_{{O}_{2}}$ (in all cases, left: 54%, UV: 71%, and right: 53%), as well as ${p}_{C{O}_{2}}$ (in all cases, left and right: 43 mmHg and UV: 36 mmHg).

**Table 6:** The simulated ${s}_{{O}_{2}}$ and ${p}_{C{O}_{2}}$ of the outflow tracts of the fetal left and right heart side and umbilical vein for the control, VSD, and TGA case

	Control		VSD		TGA	
	${s}_{{O}_{2}}$ [%]	${p}_{C{O}_{2}}$[mmHg]	${s}_{{O}_{2}}$ [%]	${p}_{C{O}_{2}}$[mmHg]	${s}_{{O}_{2}}$ [%]	${p}_{C{O}_{2}}$[mmHg]
**Left heart side**	54	43	53	43	55	42
**UV**	71	36	71	36	71	36
**Right heart side**	53	43	53	43	52	44

### Fetal-to-neonatal transition

This section demonstrates the hemodynamic impact of VSD or TGA on the fetal-to-neonatal transition, including oxygenation levels.

#### Cardiac output

**Fig. 3:**
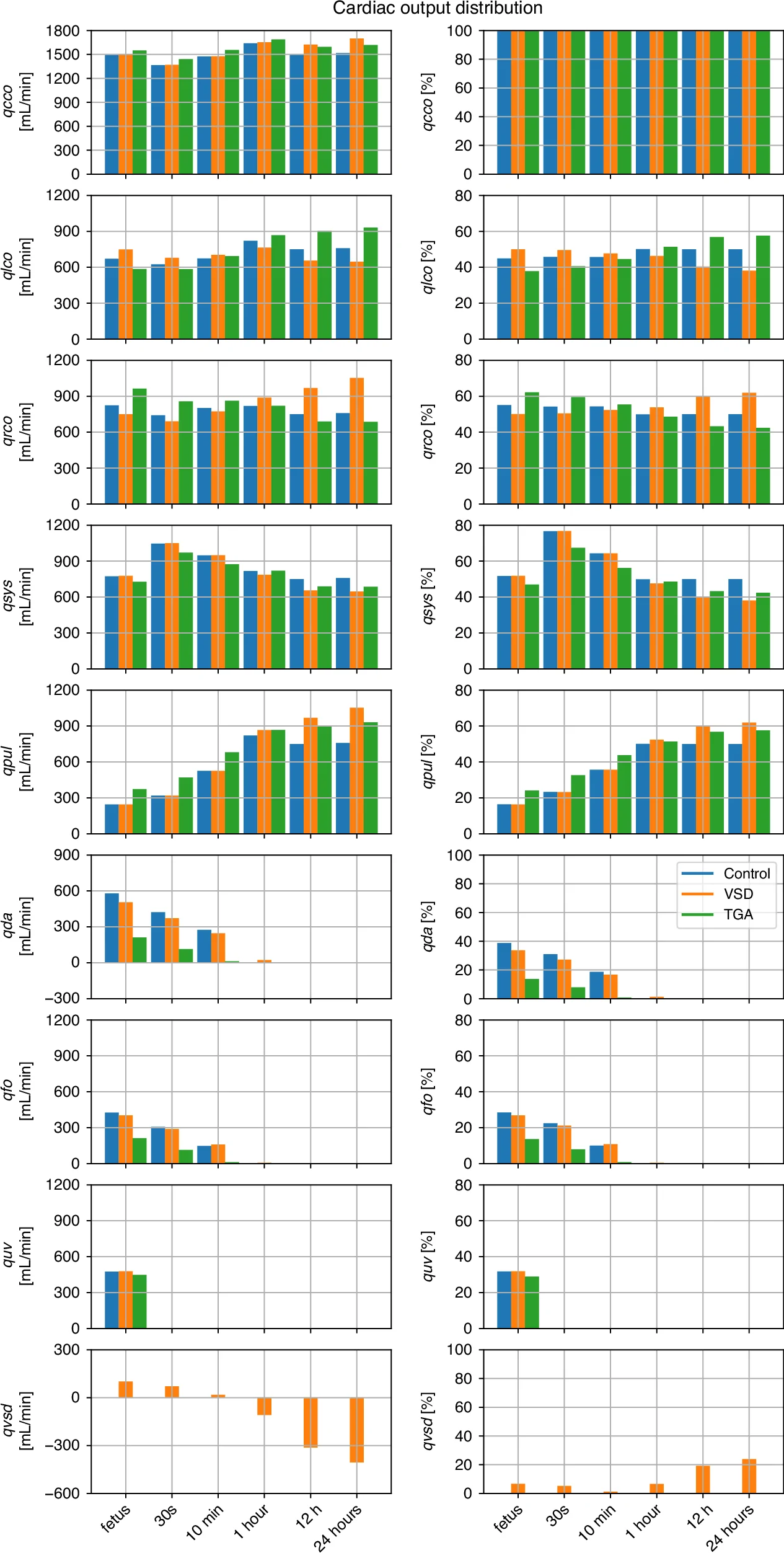
The simulated cardiac output distribution of the fetus in transition to a neonate. Absolute flow values (left) and percentage of combined cardiac output (right) are presented for cases without CHD (blue), with VSD (orange), and with TGA (green) and percentage of combined cardiac output (right). ${{{\rm{q}}}}_{{{\rm{cco}}}}$: combined cardiac output flow, ${{{\rm{q}}}}_{{{\rm{lco}}}}$: left cardiac output flow, ${{{\rm{q}}}}_{{{\rm{rco}}}}$: right cardiac output flow, ${{{\rm{q}}}}_{{{\rm{sys}}}}$: systemic circulation flow, ${{{\rm{q}}}}_{{{\rm{pul}}}}$: pulmonary circulation flow, ${{{\rm{q}}}}_{{{\rm{da}}}}$: ductus arteriosus flow, ${{{\rm{q}}}}_{{{\rm{fo}}}}$: foramen ovale flow, ${{{\rm{q}}}}_{{{\rm{uv}}}}$: umbilical vein flow, and ${{{\rm{q}}}}_{{{\rm{vsd}}}}$: flow through the VSD.

For all cases, ${q}_{{cco}}$ decreases within the first 30 s (Control: 1495–1366 mL/min, VSD: 1499 to 1369 mL/min, TGA: 1550 to 1442 mL/min), followed by an increase within the first hour (Control: 1639 mL/min, VSD: 1652 mL/min, TGA: 1687 mL/min). After an hour, ${q}_{{cco}}$ of the control and TGA cases slightly decline to 1517 and 1618 mL/min, respectively, while the VSD case increases to 1700 mL/min at 24 h after birth (Fig. 3).

The reduction in ${R}_{{pul}}$ and the closure of the ductus arteriosus during the fetal-to-neonatal transition alter both the distribution of cardiac output and the pressure balance between ventricles. In the control case, the initially right-dominant fetus transitions into an equally dominant neonate; $q{rco}/q{lco}$ reduces from 1.23 to 1.0, respectively, leading to an equally balanced flow to the pulmonary and systemic circulation (${q}_{{pul}}={q}_{{sys}}=50 \%$ of ${q}_{{cco}}$, Fig. 3). In the VSD case, the opposite effect occurs. The decline in ${R}_{{pul}}$ and the closure of the ductus arteriosus result in a net redirection of mean blood flow through the bidirectional shunt VSD (from net right-to-left to net left-to-right flow, see ${q}_{{vsd}}$ in Fig. 5), accompanied by an augmentation in mean flow (fetus: 101 mL/min and 6.8% of ${q}_{{cco}}$, neonate 24 h: –406 mL/min and 23.9% of ${q}_{{cco}}$). Consequently, this shunt initially results in a ${q}_{{pul}}$ matching the control case at 16% of ${q}_{{cco}}$, but increases after birth to 62% of ${q}_{{cco}}$, deviating from the equally balanced distribution between pulmonary and systemic flow observed in the control case (Fig. 3). In the TGA case, fetal ${q}_{{rco}}$ is higher compared to the control case. This could be due to the structural difference represented in the TGA model, including a larger tricuspid valve area and a smaller mitral valve area, as reported by Godfrey et al.^7^ and implemented in our model accordingly (Table 1). However, as the transition progresses, ${q}_{{rco}}$ reaches approximately similar values as the control case, leading ${q}_{{rco}}/{q}_{{lco}}$ = 0.74. Hence, the TGA case switches from a right-dominant to a left-dominant heart, like the control case. However, the pressure difference between the ventricles is during the neonatal phase not as large compared to the control case.

#### Shunts

The umbilical cord clamping at 30 s after birth interrupts the placental circulation and subsequently umbilical vein flow ${q}_{{uv}}$ is ceased (Fig. 3), triggering ductus arteriosus constriction and diminishing of ${q}_{{da}}$. Notably, ${q}_{{da}}$ first reverses before diminishing (Fig. 5). This pattern is consistent across all three cases. The flow ${q}_{{fo}}$ diminishes as the transition progresses due to the changes in pressure gradient between the left ${p}_{{la}}$ and right ${p}_{{ra}}$ atrium (Fig. 6). This diminishing is seen in all cases.

#### E/A ratio

Figure 5 illustrates the flows through the mitral ${q}_{{mv}}$ and tricuspid ${q}_{{tv}}$ valves, as well as flow through the semilunar valves leaving the left ${q}_{{lco}}$ and right ${q}_{{rco}}$ ventricles. In the control case, as the transition progresses, passive filling (E-peak) surpasses active filling (A-peak) in the left side of the heart (Control ${E}_{{mv}}$/${A}_{{mv}}$ fetus: 0.77, neonate 24 h: 1.20), while the E/A ratio in the tricuspid valve remains relatively stable (Control ${E}_{{tv}}$/${A}_{{tv}}$ fetus: 0.77, neonate 24 h: 0.75). In the TGA and VSD case, similar trends are visible, with the ${E}_{{mv}}$/${A}_{{mv}}$ increasing from fetus to neonatal physiology and ${E}_{{tv}}$/${A}_{{tv}}$ remaining stable (TGA: ${E}_{{mv}}$/${A}_{{mv}}$ fetus: 0.73, neonate 24 h: 1.19 and ${E}_{{tv}}$/${A}_{{tv}}$ fetus: 0.84, neonate 24 h: 0.81). However, the VSD circulation shows a less pronounced elevation of ${E}_{{mv}}$/${A}_{{mv}}$ (VSD: ${E}_{{mv}}$/${A}_{{mv}}$ fetus: 0.75, neonate 24 h: 0.90 and ${E}_{{tv}}$/${A}_{{tv}}$ fetus: 0.79, neonate 24 h: 0.79).

#### Pulmonary circulation

Lung aeration induces a rapid decrease in ${R}_{{pul}}$, leading to higher ${q}_{{pul}}$ (Fig. 5) and increased venous return. In the control case, this elevation, coupled with shunt closure, results in a rise in mean ${p}_{{lv}}$ from 26 mmHg before birth to 40 mmHg after 24 h, while the mean ${p}_{{rv}}$ decreases from 29 to 26 mmHg (Fig. 6). This trend does not hold for the CHD cases. The net left-to-right shunting through the VSD reduces the pressure difference between the ventricles and increases ${q}_{{mpa}}$ and ${p}_{{mpa}}$, while decreasing mean ${p}_{{asc}}$ compared to control case (Fig. 6). In the TGA case, both mean ventricular pressures rise (${p}_{{lv}}$: 25–37 mmHg and ${p}_{{rv}}$: 30–32 mmHg). The initially higher ${q}_{{pul}}$ achieves similar values compared to control case around 24 h as ${R}_{{pul}}$ equals that of other cases.

### Blood gases

**Fig. 4:**
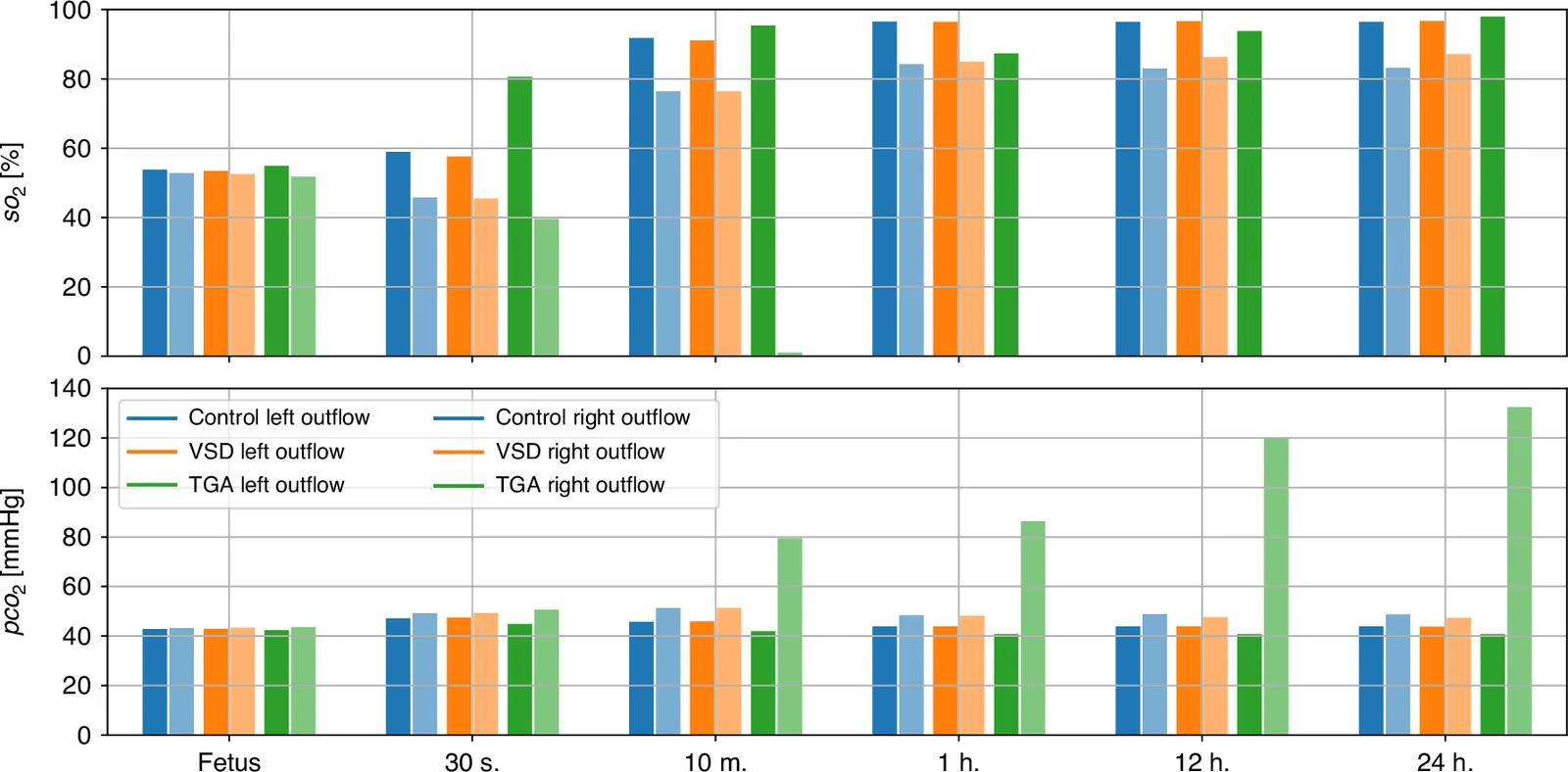
${{{\rm{s}}}}_{{{{\rm{O}}}}_{2}}$ (upper) and ${{{\rm{p}}}}_{{{\rm{C}}}{{{\rm{O}}}}_{2}}$ ${{{\rm{p}}}}_{{{\rm{C}}}{{{\rm{O}}}}_{2}}$ (lower) during fetal-to-neonatal transition of the left (darker color) and right (lighter color) outflow tract of the heart for the control (blue), VSD (orange), and TGA (green) case.

Figure 4 illustrates ${s}_{{O}_{2}}$ and ${p}_{C{O}_{2}}$ in the outflow vessels from the left and right sides of the heart. In the control case, within the first 30 s after birth, ${s}_{{O}_{2}}$ in the ascending aorta (left outflow) increases to 59%, while it decreases to 46% in the main pulmonary artery (right outflow). After 10 min, ${s}_{{O}_{2}}$ rises to 92% and 83%, respectively, and eventually reaches 96% and 83% after 12 h. The ${p}_{C{O}_{2}}$ in the aorta remains approximately 43 mmHg, while in the main pulmonary artery, it increases from 43 to 49 mmHg. The VSD case follows a similar trend; however, ${s}_{{O}_{2}}$ in the main pulmonary artery is higher (87%) at neonatal endpoint. In the TGA case, ${s}_{{O}_{2}}$ in the ascending aorta (right outflow) decreases to zero after 10 min, while ${p}_{C{O}_{2}}$ increases to 132 mmHg.

## Discussion

This study demonstrates the ability of a closed-loop 0D-1D fetal cardiovascular model, including the one-fiber model, to simulate the hemodynamic effects of VSD and TGA during fetal-to-neonatal transition. In addition, it offers insights into hemodynamic changes occurring during this transition, particularly concerning CHDs. The model can serve as a foundation for various applications in perinatal hemodynamics research, including studies on the fetal-to-neonatal transition. The following sections discuss major findings and limitations.

### Full-term fetus

Table 5 shows that the simulated cardiac output distribution aligns with literature data.^6,48^ Compared to the control case, ${q}_{{lco}}$ and ${q}_{{rco}}$, resulting from pathological cases, show a discrepancy due to the extra shunt (VSD) or the reduced pulmonary resistance (TGA) (Fig. 6). Despite these deviations, the fetal shunts provide compensation, ensuring the maintenance of organ perfusion. Blood flow fractions to the pulmonary, systemic, and placental circulation (${q}_{{pul}}$, ${q}_{{sys}}$, and ${q}_{{uv}}$, respectively) remain consistent between control and VSD case, agreeing with existing literature.^14^ The maximal difference observed between control and TGA case is only 8% (Table 5). As organ perfusion remains sustained in fetuses with CHD due to fetal shunts, also ${s}_{{O}_{2}}$ and ${p}_{C{O}_{2}}$ levels are maintained (Table 6). Saini et al.^49^ report umbilical artery (UA) and vein (UV) values (UA: ${s}_{{O}_{2}}$ = 50% and ${p}_{C{O}_{2}}$ = 52 mmHg, UV: ${s}_{{O}_{2}}$= 70% and ${p}_{C{O}_{2}}$ = 30 mmHg), aligning with simulations of this study (estimated with the outflow tracts UA: ${s}_{{O}_{2}}$ = 54% and ${p}_{C{O}_{2}}$ = 43 mmHg and UV: ${s}_{{O}_{2}}$ = 71% and ${p}_{C{O}_{2}}$ = 36 mmHg).

To verify the simulated reduction of 43% of ${R}_{{pul}}$ in the fetal circulation with TGA compared to control case, the increase in ${q}_{{pul}}$ and decrease in ${q}_{{da}}$ due to this resistance reduction is compared with Lachaud et al.^6^ and Charbonneau et al.^46^. The simulated values agree with reported values in literature (Table 7).

**Table 7:** Comparison of simulated pulmonary ${q}_{{pul}}$ and ductus arteriosus ${q}_{{da}}$ flow with literature data.^6,47^

Parameter	Simulated flow [mL/min]	Lachaud et al.^6^ (mean ± standard deviation)	Charbonneau et al.^47^ (median [interquartile range])
${q}_{{pul}}$	375	416 ± 238	344 [272 467]
${q}_{{da}}$	212	155 ± 117	164 [43 251]

**Fig. 5:**
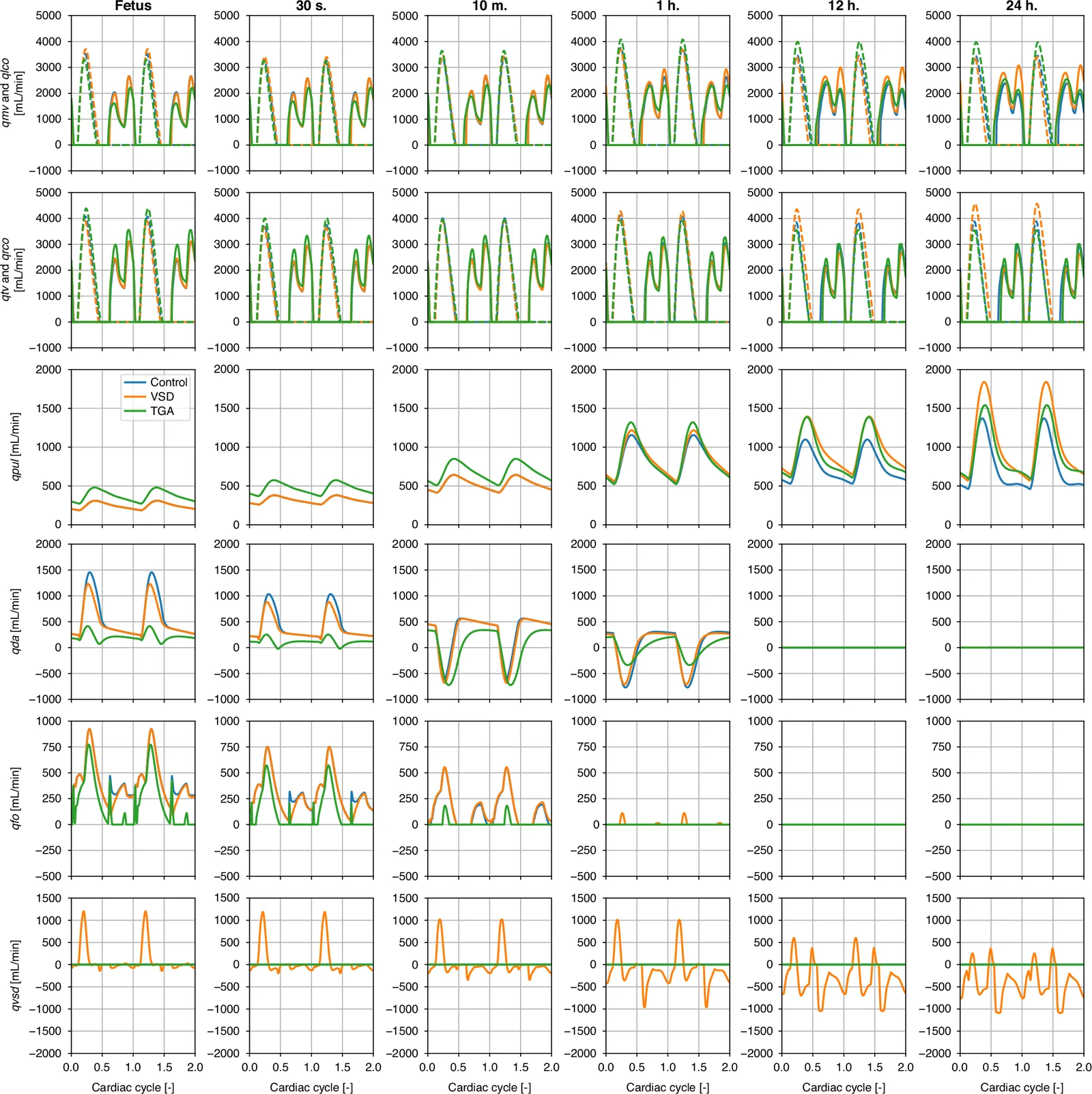
Simulated flow profiles of a fetus transitioning into a neonate without CHD (blue), with VSD (orange), and with TGA (green). ${{{\rm{q}}}}_{{{\rm{mv}}}}$: mitral valve flow, ${{{\rm{q}}}}_{{{\rm{lco}}}}$: flow leaving left ventricle (dashed), ${{{\rm{q}}}}_{{{\rm{tv}}}}$: tricuspid valve flow, ${{{\rm{q}}}}_{{{\rm{rco}}}}$: flow leaving left ventricle (dashed), ${{{\rm{q}}}}_{{{\rm{pul}}}}$: pulmonary circulation flow, ${{{\rm{q}}}}_{{{\rm{da}}}}$: ductus arteriosus flow, ${{{\rm{q}}}}_{{{\rm{fo}}}}$: foramen ovale flow, and ${{{\rm{q}}}}_{{{\rm{vsd}}}}$: flow through the VSD.

Besides the unique shunts, typical for the fetal circulation is that the mitral (${E}_{{mv}}/{A}_{{mv}}$) and tricuspid valve (${E}_{{tv}}/{A}_{{tv}}$) E/A ratio have a smaller value than 1.0,^50,51^ matching our simulations (${E}_{{mv}}/{A}_{{mv}}$ = ${E}_{{tv}}/{A}_{{tv}}$ = 0.77) (Fig. 5). The VSD and TGA cases show similar results to the control case (Fig. 5). However, the ${E}_{{tv}}/{A}_{{tv}}$ is higher for the TGA case (${E}_{{tv}}/{A}_{{tv}}$ = 0.84), indicating larger right ventricular inflow due to passive filling, which is caused by a larger ${q}_{{pul}}$ due to lower ${R}_{{pul}}$.

These results demonstrate that the mathematical model can realistically simulate the hemodynamics of a full-term fetus without CHD, with VSD, and with TGA in comparison to literature data. In addition, it highlights the compensatory capacity of the fetal shunts in maintaining organ perfusion during CHDs, emphasizing the challenge in predicting the postnatal impact of CHDs when only flow measurements are available prenatally.

### Fetal-to-neonatal transition

**Fig. 6:**
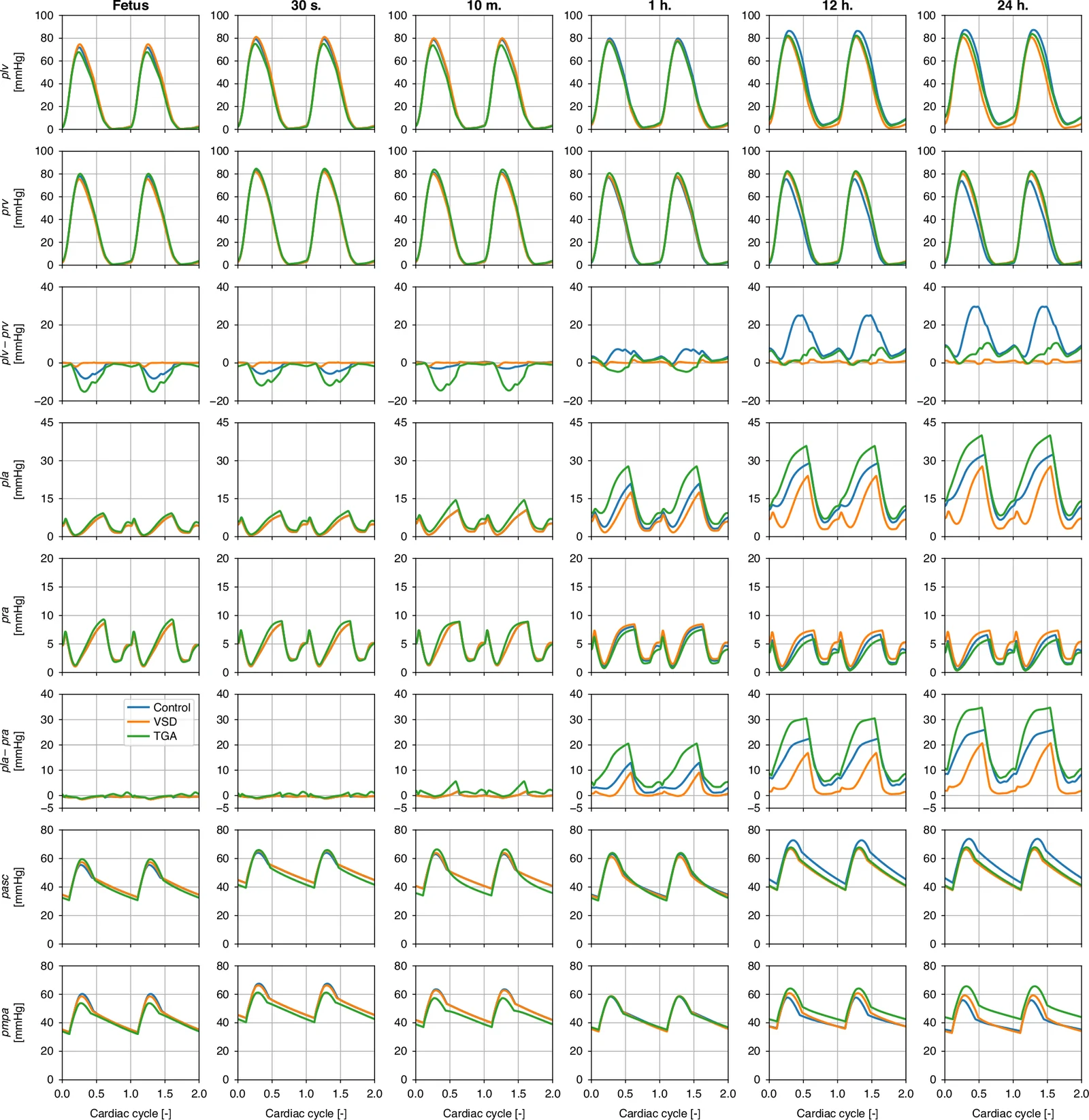
Simulated pressure profiles of a fetus in transitioning to a neonate without CHD (blue), with VSD (orange), and with TGA (green). ${{{\rm{p}}}}_{{{\rm{lv}}}}$: left ventricular pressure, ${{{\rm{p}}}}_{{{\rm{rv}}}}$: right ventricular pressure, ${{{\rm{p}}}}_{{{\rm{lv}}}}-{{{\rm{p}}}}_{{{\rm{rv}}}}$: pressure difference between left and right ventricle, ${{{\rm{p}}}}_{{{\rm{la}}}}$: left atrial pressure, ${{{\rm{p}}}}_{{{\rm{ra}}}}$: right atrial pressure, ${{{\rm{p}}}}_{{{\rm{ra}}}}-{{{\rm{p}}}}_{{{\rm{la}}}}$: pressure difference between right and left atrium, ${{{\rm{p}}}}_{{{\rm{asc}}}}$: ascending aorta pressure, and ${{{\rm{p}}}}_{{{\rm{mpa}}}}$: main pulmonary artery pressure.

Figures 3– 6 demonstrate the impact of cardiovascular adaptations occurring during fetal-to-neonatal transition, simulated by changes in resistances, compliances, and diffusive conductances (Fig. S.1), on fetuses without CHD, with VSD, and with TGA.

#### Control

Once the umbilical cord has been clamped, there is a notable decrease in ${q}_{{cco}}$ simulation results across all cases (<30 s). Subsequently, there is a rise in ${q}_{{cco}}$ within the first hour, primarily driven by the ${q}_{{lco}}$, due to an increase in ${C}_{{pul},a}$ and ${C}_{{pul},v}$ and a reduction in ${R}_{{pul}}$ (Fig. 3). This is consistent with the study of Vonderen et al.^38^ which reported a maintained ${q}_{{rco}}$ from 2 to 10 min after birth and an increase in ${q}_{{lco}}$. After an hour, ${R}_{{sys}}$ increases resulting in a decrease of ${q}_{{lco}}$, aligning with the observations of Agata et al.^52^

Another observation, is the increase in ${E}_{{mv}}/{A}_{{mv}}$ ratio (fetus: 0.77, neonate 24 h: 1.20), while ${E}_{{tv}}/{A}_{{tv}}$ ratio remains relatively stable (fetus: 0.77, neonate 24 h: 0.75) as the transition progresses. This aligns with literature findings, indicating an elevated ${E}_{{mv}}/{A}_{{mv}}$ ratio and a relatively constant ${E}_{{tv}}/{A}_{{tv}}$ ratio.^53^

Furthermore, the simulated systolic ${p}_{{mpa}}$ decreases from 67.5 at 30 s. to 57.8 mmHg at 12 h. after birth, which agrees with Kang et al.^54^ (79.2 ± 6.5 mmHg for males and 74.4 ±7.2 mmHg for females at <2 h; 52.8 ± 8.96 mmHg for males and 52.17 ± 9.32 mmHg for females at 12 h.) and Hu et al.^55^ (62.6 $\pm \,7.6$ mmHg at 0–6 h.). Simulated mean and diastolic ${p}_{{mpa}}$ drop from 53.6 at 30 s. to 44.1 mmHg at 12 h. and 43.2 at 30 s. to 34.2 mmHg at 12 h., respectively. These values are higher compared to the data of Kang et al., though they report no standard deviations for mean and diastolic pressures. Given the variability in systolic pressure, the simulated values likely fall within physiological range. Still, pulmonary artery compliance in the model may be underestimated.

Besides the blood circulation model, the blood gas model also simulates realistic values during the fetal-to-neonatal transition. At 10 min, the simulated ${s}_{{O}_{2}}$ in the ascending aorta (left outflow tract) and main pulmonary artery (right outflow tract) is 92% and 83%, respectively, at 10 min. Mariani et al.^56^ reported a pre-ductal ${s}_{{O}_{2}}$ of 96% at 10 min after birth. Furthermore, the simulated ${p}_{C{O}_{2}}$ remains stable around 43 mmHg, within clinically accepted ranges (37.5 mmHg to 52.5 mmHg).^57^

These results suggest that the model can realistically simulate the healthy fetal-to-neonatal transition.

#### Ventricular septal defect

Typically, VSDs show bidirectional shunting with minimal net flow due to approximately equal ventricular pressures. Our model produces a net right-to-left flow during the fetal phase and a net left-to-right flow in the neonatal phase. The VSD is not only determined by the pressure gradient between the ventricles, but also by pulmonary resistance; an overestimation and underestimation, respectively, of resistance in the tricuspid valve, pulmonary trunk, or pulmonary microcirculation may explain the observed net flow direction.

Another observation is that ${q}_{{mv}}$ is increased compared to the control case during neonatal phase, reflecting the higher pulmonary return due to left-to-right shunting. However, ${q}_{{lco}}$ remains unchanged, because part of the blood entering the left ventricle is redirected through the VSD into the right ventricle instead of being ejected into the aorta.

In contrast to the control case, ${q}_{{cco}}$ resulting from the circulation with VSD solely increases after removal of the placental circulation primarily driven by ${q}_{{rco}}$ (Fig. 5), agreeing with the study of Rudolph.^14^ This increase in ${q}_{{rco}}$ and, thus, an elevated ${q}_{{pul}}$ leads to a rise in simulated ${p}_{{rv}}$ after 12 h compared to the control case (Fig. 6). While a VSD does not typically require immediate clinical intervention, it serves as a proof-of-concept to evaluate how structural defects affect flow redistribution. In our model, the left-to-right shunt after birth leads to increased pulmonary flow and thus to increased ${q}_{{rco}}$, as the right ventricle ejects both systemic venous return and shunted volume.

Despite the increased ${q}_{{rco}}$, the pressure gradient between left and right ventricle remains unchanged compared to the control case, suggesting that right ventricular pressure is not increased. This aligns with clinical understanding that VSD shunting primarily causes volume overload of the left atrium and ventricle, as also described by Penny et al.^58^. Hence, depending on the amount of inter-ventricular shunting, the increase in ${q}_{{pul}}$ may lead to complications, such as pulmonary hypertension that ultimately could lead to right ventricular hypertrophy.^58^ Therefore, symptoms may manifest later in life, making an accurate assessment of VSD crucial. However, such assessments during pregnancy using 2D Doppler are not sufficiently accurate due to Doppler angle dependency and geometric assumptions. Since ${q}_{{fo}}$ and ${q}_{{da}}$ are affected by VSD, measuring these two flows relative to$\,{q}_{{uv}}$, in combination with model predictions of postnatal ${s}_{{O}_{2}}$, may improve fetal diagnosis and understanding of the postnatal impact of VSD.

#### Transposition of the great arteries

In TGA, the right ventricle supports the systemic circulation after birth and is therefore expected to generate higher pressures than the left ventricle. This expectation is mainly based on physiological reasoning and experimental data as described by Rudolph.^14^ Still, the magnitude of this pressure difference in neonates is uncertain. In our model, the difference is attenuated, possibly due to altered resistance distributions, such as decreased mitral and increased tricuspid valve resistance.

Neonates with TGA face immediate postnatal challenges due to complete separation of systemic and pulmonary circulations, resulting in severe hypoxia as oxygen-rich blood in the pulmonary circulation fails to reach the systemic circulation.^14,44^ This is evident in Fig. 4, where ${s}_{{O}_{2}}$ in the main pulmonary artery (left outflow tract) is 95% after 10 min and increases to 98% within 24 h, while in the ascending aorta (right outflow tract), saturation drops to 1% and eventually to 0%, respectively. In addition, ${p}_{C{O}_{2}}$ in the ascending aorta increases even to 132 mmHg within 24 h. A case study presented by Hoshino et al.^59^ shows a ${p}_{C{O}_{2}}$ of 139 mmHg upon arrival at the neonatal intensive care unit. The fast decline and rise of ${s}_{{O}_{2}}$ and ${p}_{C{O}_{2}}$ require immediate treatment after birth. The degree and duration of hypoxia predicts neurocognitive deficits later in life.^60^ Consequently, clinical management of newborns with TGA focuses on mitigating hypoxia by promoting inter-circulatory mixing through the forced patency of the ductus arteriosus and foramen ovale.^61^

However, the condition is rarely that straightforward and many infants survive beyond the 10 min. 40–45% of infants with TGA have a VSD,^62^ which allows for some degree of inter-circulatory mixing. Even in the absence of a VSD, most infants survive beyond 10 min due to fetal shunts that remain patent in response to low oxygen levels.^63^ By structurally analyzing the fetal heart, TGA patients, with or without VSD, can be identified. This modeling approach holds promise for treatment planning by providing insight into the impact of VSD and/or maintaining the ductus arteriosus and foramen ovale patency postnatally. Additionally, it allows for the optimization of shunt diameters to enhance inter-circulatory mixing. Personalizing the model with individual ultrasound measurements may improve prenatal risk stratification and aid in identifying fetuses who could benefit from early interventions, such as the Rashkind maneuver.^64^

### Limitations

The model simulations in this study rely on predefined parameter adaptations to mimic fetal-to-neonatal transition. While this approach results in realistic hemodynamic behavior, these adaptations are influenced by various factors including blood gas content, hormones, flows, and pressures.^65,66^ This predefined trend of parameters simplifies the fetal-to-neonatal transition, potentially overlooking nuances in the hemodynamics.

The cardiac valves are modeled as diodes, connected to large vessels described by a 1D model. Consequently, the valve resistance must equal the characteristic impedance of the corresponding vessel. This leads to relatively high valve resistances and pressure drops over the semilunar valves, resulting in supraphysiological ventricular pressures. In addition, the assumption of uni-directional foramen ovale flow may oversimplify its complex behavior, particularly in cases of bi-directional flow. For instance, Godfrey et al.^7^ found that 75.7% of fetuses with TGA have bi-directional foramen ovale flow. Figure 6 illustrates a pressure drop from left to right (${p}_{{la}}-\,{p}_{{ra}}$), which is likely to lead to a left-to-right foramen ovale flow if a bi-directional valve were used. In instances of bi-directional flow, this model would overestimate pulmonary flow. Improving the model’s accuracy may involve investigating better valve representations.

Table 6 shows a minimal difference in ${s}_{{O}_{2}}$ between the left and right outflow tracts for the full-term control case. In contrast, Rudolph^14^ reports a higher ${s}_{{O}_{2}}$ in the left ventricle (65%) compared to the right (55%). In the oxygen transport model used in this study, oxygen-rich blood from the ductus venosus is mixed with oxygen-poor blood from the superior vena cava. However, in reality, much of this oxygen-rich blood is directed almost exclusively to the left atrium with minimal mixing. As a result, the model underestimates the ${s}_{{O}_{2}}$ of the left side of the heart.

### Outlook and future research

This model has the potential to significantly aid treatment planning by estimating the hemodynamic impact of CHDs on neonatal outcomes prenatally. To fully realize this potential, it is crucial to identify and accurately parameterize the factors that most influence simulation results (i.e. sensitivity and uncertainty analysis).^67^ Prospective experimental validation is outside the scope of this study; however, future validation should integrate these influential factors with fetal measurement data, such as Doppler ultrasound velocity profiles in shunts (e.g. foramen ovale and ductus arteriosus) and major vessels (e.g. aorta, umbilical vessels, and pulmonary vessels). While beat-to-beat intervals can be obtained with Doppler ultrasound, non-invasive fetal electrocardiograms, though not widely accessible, could offer higher-quality signals for analyzing parameters such as heart rate variability and rhythm patterns, thereby further personalizing the model. By coupling clinical measurements with the model described in this study, it is possible to develop a patient-specific model that could enhance clinical decision-making. This study provides a mathematical foundation for investigating the fetal-to-neonatal transition, including the hemodynamics of CHDs. The next step is to adapt the model to individual patients, thereby further improving clinical decision-making.

## Conclusion

This study presents a novel 0D-1D closed-loop fetal mathematical model including blood gas exchange and fetal-to-neonatal transition to investigate the hemodynamic impact of two CHDs: VSD and TGA. While these diseases have minor impact during fetal stage due to the compensatory function of the fetal shunts, their hemodynamic and blood gas exchange consequences become more pronounced during early neonatal life. Hence, predicting the severity of these diseases during the prenatal period is challenging due to limited fetal impact and scarce data acquisition. However, the mathematical model proposed in this study provides potentially better understanding of the hemodynamical changes in fetal-to-neonatal transition affected by CHDs. Therefore, the model could support clinical decision making and clinical intervention time management based on prenatal diagnosis.

## Supplementary information

Supplementary information

## Data Availability

Data sharing is not applicable for this article as no new data were created or analysed in this study.
