# Supplementary material for: The hemodynamic impact of congenital heart diseases during fetal-to-neonatal transition: an in-silico investigation
Source: Pediatr Res. 2026 Jan 30;100(3):1056–68. doi: 10.1038/s41390-025-04565-1 (PMC13672090; doi:10.1038/s41390-025-04565-1)
Supplement: Supplementary file 1 [file 41390_2025_4565_MOESM1_ESM.pdf]

Supplementary Material

S. Fetal-to-neonatal transition

This supplementary material describes the changes of the fetal shunts and pulmonary and systemic circulation during fetal-to-neonatal transition. Figure S.1 provides an overview of these adaptations relative to their fetal values (denoted with subscript 0) or neonatal endpoint values (denoted with subscript 1). This section briefly describes the changes in parameters. Table S.1 gives an overview of all parameter values.

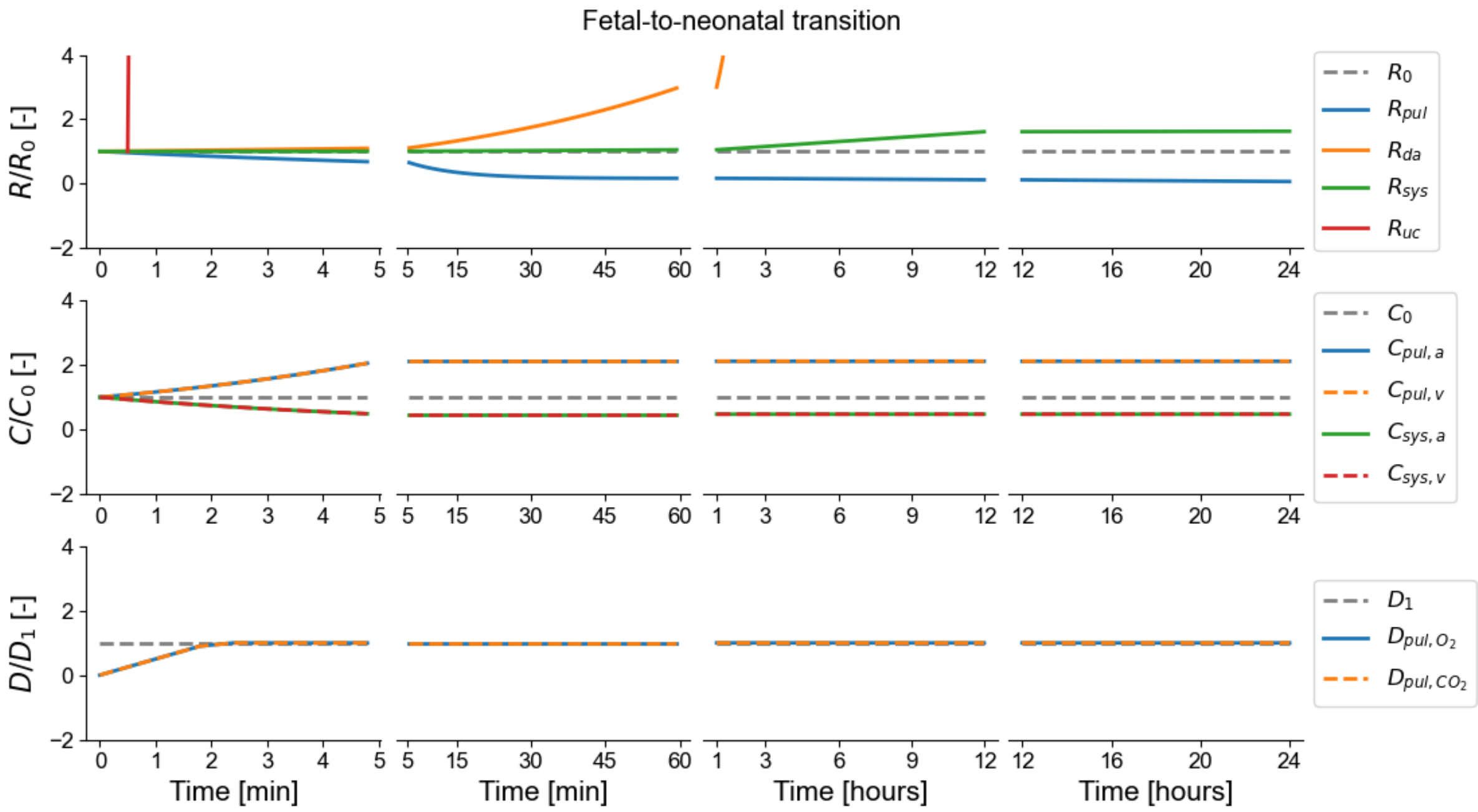

Figure S.1:Adaptation of parameters describing fetal physiology from transition to neonatal physiology relative to their fetal resistance or compliance value (R_0 or C_0), respectively, or neonatal diffuse conductance value (D_1). R_{uc}: umbilical cord resistances, R_{pul}: pulmonary resistance, R_{da}: ductus arteriosus resistance, R_{sys}: systemic resistance, R_{uv}: umbilical vein resistance, $C_{pul,a}$: pulmonary arterial compliance, $C_{pul,v}$: pulmonary venous compliance, D_{pul,O_2}: oxygen diffuse conductance of the lungs, and D_{pul,CO_2}: carbon dioxide diffuse conductance of the lungs.

S.1 Placental circulation

Shortly after birth, the umbilical cord clamping is simulated by instantaneously elevating the umbilical arterial (R_{ua}) and umbilical venous resistance (R_{uv}) to a value of $\mathcal{O}(10^{39})$ mmHg s/mL at 30 seconds after birth (in Figure S.1 denoted as R_{uc}).

S.2 Ductus arteriosus

The constriction of the ductus arteriosus is related to the rise in blood oxygen tension and the termination of placental functioning, and thus the loss of a major source for prostaglandin E_2

(1). The closure of the ductus arteriosus is based on literature studies (2–4), and can be modeled by the following function:

$$r_{da}(t) = \frac{1}{2} r_{da,0}(e^{m_{da,1}t} + e^{m_{da,2}t}), \quad (1)$$

where $m_{da,1}$ and $m_{da,2}$ are scaling constants, t is the time in hours, and $r_{da,0}$ is the radius of the ductus arteriosus just before birth. The resistance of the ductus arteriosus is then calculated by:

$$R_{da}(t) = \frac{K_{da}}{r_{da}^4(t)}, \quad (2)$$

where K_{da} is chosen, such that $R_{da}(t = 0) = R_{da,0}$ with $R_{da,0}$ the fetal ductus arteriosus resistance.

The ductus arteriosus diameter for fetuses with TGA is smaller compared to healthy fetuses (5,6). Therefore, for the TGA case, the fetal ductus resistance $R_{da,0}$ is adjusted to match a diameter of 3.6 mm (6).

S.3 Foramen ovale

Following cord clamping, lung aeration occurs, leading to a shift in pressure drop between the atria and subsequently closure of the foramen ovale, as this valve is described as a right-to-left uni-directional valve (Equation 2). The closure of the foramen ovale is a model result rather than a prescribed function like the ductus arteriosus (Figure S.1).

S.4 Lung aeration

As lung aeration progresses, the conductance of oxygen and carbon dioxide of the lungs (D_{pul,O_2} and D_{pul,CO_2}, respectively) linearly increases until full aeration is achieved after approximately 2 minutes (7). At start of birth ($t_0 = 0$), both diffusive conductances increase linearly with the time until the lungs are fully aerated (2):

$$D_{pul,O_2}(t) = \begin{cases} 0 & t < t\_0 \\ D_{pul,1}\frac{(t-t_0)}{t_{d,1}} & t_0 < t \le t_{d,1}, \\ D_{pul,1} & t > t_{d,1} \end{cases} \quad D_{pul} = \{D_{pul,O_2}, D_{pul,CO_2}\}, \quad (3)$$

where $t_{d,1}$ is the duration of achieving fully aerated lungs and $D_{pul,1} = \{D_{pul,O_2,1}, D_{pul,CO_2,1}\}$ is the diffusive conductance at full aeration. The parameter $t_{d,1}$ is set on 2 minutes, as this is the time it takes to achieve normal neonatal tidal volume and maximum exhaled CO_2 (7,8).

The process of lung aeration causes a decreased pulmonary vascular resistance (R_{pul}), which leads to an increased pulmonary blood flow. The time-varying R_{pul} is prescribed by a rapid decrease for the first 36 minutes after birth, followed by a more gradual linear decrease afterwards (2). This rapid decrease is based on fetal lamb data, which showed a rapid 74\% pulmonary vascular resistance decrease in the first 36 minutes after ventilation (9). The mathematical formulation is based on the study of Sá-Couto et al., followed by adaptation to result a neonatal end value of 0.5 mmHg s/mL, agreeing with Yigit et al. (10):

$$R_{pul}(t) = \begin{cases} R_{(pul,0)}\left(1 - m_{(pul,1)}(1 - e^{m_{(pul,2)}t})\right) & t_0 \leq t \leq t_{pul,1} \\ R_{(pul,0)}\left(1 - m_{(pul,1)}(1 - e^{m_{(pul,2)}t}) - m_{(pul,3)}\left(\frac{t - t_{pul,1}}{t_{pul,1}}\right)\right) & t_{pul,1} < t \leq t_1 \end{cases}$$

where $R_{(pul,0)}$ is the pulmonary vascular resistance for a fetus just before birth ($t_0 = 0$), $t_{pul,1}$ are time constants in hours, t_1 = 24 hours, and $m_{(pul,1)}$, $m_{(pul,2)}$, and $m_{(pul,3)}$ are scaling constants.

In fetuses with TGA, a high oxygen-saturated left ventricular blood flows through the pulmonary artery, resulting in a lower pulmonary vascular resistance (5,11,12). The blood flow to the pulmonary vascular bed is 56\% higher (12). Based on this reported value, a fetal pulmonary vascular resistance $R_{(pul,0)}$ is calculated for fetuses with TGA. The scaling parameters $m_{(pul,1)}$ and $m_{(pul,3)}$ are adapted such that neonatal endpoint is equal to the control case.

At birth, the newborn needs to clear the airway liquid rapidly to allow the onset of pulmonary gas exchange (13). The process of airway liquid clearance is believed to change the lung compliance, the ability of the lungs to expand. Furthermore, during the transition, there are several other factors that could possibly affect the pulmonary compliance, such as an increased number of active capillaries contributing to the organ vascular compliance, changes in structural stiffness due to lung volume variations and strain-dependent elasticity of the lung parenchymal tissue, mechanical instabilities, vasodilation, changes in surface tension at the air-liquid interface, and short term vascular remodeling that follows the expansion (10). Clinical data on fetal lamb suggested an exponential twofold increase in lung compliance in the first five minutes after birth, after that the compliance remains fairly constant (14). This literature data is used to define an exponential increase for the first five minutes after birth and constant value in the successive 24 hours:

$$C_{pul}(t) = \begin{cases} C_{pul,0}e^{m_{c,1}t} & t_0 \leq t \leq t_{c,1} \\ C_{pul,0}e^{m_{c,1}t_{c,1}} & t_{c,1} < t \leq t_1 \end{cases}, \quad C_{pul} = \{C_{pul,a}, C_{pul,v}\}, \tag{4}$$

where $m_{c,1}$ is the shape constant, $t_{c,1}$ is the time constant, and $C_{pul,0} = \{C_{pul,a,0}, C_{pul,v,0}\}$ is the fetal compliance of the pulmonary artery and the pulmonary vein just before birth. This results in an approximated twofold increase for both the compliance representative for the pulmonary artery and pulmonary vein.

S.5 Systemic circulation

With the decrease in pulmonary vascular resistance R_{pul} and the increase in pulmonary compliance C_{pul}, the systemic resistance R_{sys} increases. The increase in systemic vascular resistance is based on Sá-Couto et al. (15), which used calculated total systemic resistance values from healthy full-term fetuses (16–18) to establish a piece-wise linear function. Furthermore, Doni et al. (19) evaluated the hemodynamics in healthy term neonates using an ultrasonic cardiac output monitor which showed an initial steep increase followed by a more gradual increase. The increase of the systemic vascular resistance R_{sys} is primarily caused by the removal of the placenta, leading to a significant amount of blood loss (20). In addition, it is suggested that the release of catecholamine and the increased high sympathetic tone after

birth play an important role in the increasing systemic vascular resistance (15). The following time-dependent relation is adopted from the study of Sá-Couto et al. (15) and adapted to a neonatal end value of 3.7 mmHg s/mL, agreeing with Mynard (21):

$$R_{sys}(t) = \begin{cases} R_{sys,0} & t_0 \le t < t_{sys,1} \\ R_{sys,0}\left(1 + m_{sys,1}\left(\frac{t - t_{sys,1}}{t_{sys,1}}\right)\right) & t_{sys,1} \le t \le t_{sys,2} \\ R_{sys,0}\left(1 + m_{sys,1}\left(\frac{t - t_{sys,1}}{t_{sys,1}}\right) + m_{sys,2}\left(\frac{t - t_{sys,2}}{t_{sys,2}}\right)\right) & t_{sys,2} < t \le t_1 \end{cases}, \quad (5)$$

where $R_{sys,0}$ is the systemic vascular resistance for a full-term fetus, $t_{sys,1}$ and $t_{sys,2}$ are time constants, and $m_{sys,1}$ and $m_{sys,2}$ are scaling constants.

The high sympathetic tone decreases the systemic compliance. However, it is unknown how much. Therefore, in this study, the same exponential function as for the pulmonary compliances is chosen, only in the opposite direction:

$$C_{sys}(t) = \begin{cases} C_{sys,0}e^{-m_{c,1}t} & t_0 \le t < t_{c,1} \\ C_{sys,0}e^{-m_{c,1}t_{c,1}} & t_{c,1} < t \le t_1 \end{cases}, \; C_{sys} = \{C_{sys,a}, C_{sys,v}\}, \quad (6)$$

where $C_{sys} = \{C_{sys,a,0}, C_{sys,v,0}\}$ is the fetal compliance of the systemic atrial and venous side just before birth.

Table S.1: Transitional input parameters.

Parameter	Abbreviation	No CHD	VSD	TGA	Unit
Fetal ductus arteriosus radius	$r_{da,0}$	2.5		1.8	mm
Ductus arteriosus scaling constant	$m_{da,1}$	-0.43			hour^{-1}
Ductus arteriosus scaling constant	$m_{da,2}$	-0.14			hour^{-1}
Ductus arteriosus resistance scaling constant	K_{da}	$4.69 \cdot 10^{-4}$			mmHg s cm
Diffusive conductance time constant	$t_{d,1}$	2			min
Pulmonary resistance scaling constant	$m_{(pul,1)}$	0.838		0.82	-
Pulmonary resistance scaling constant	$m_{(pul,2)}$	-6.0			-
Pulmonary resistance scaling constant	$m_{(pul,3)}$	0.009		0.0064	-
Pulmonary resistance time constant	$t_{pul,1}$	0.6			hours
Compliance scaling constant	$m_{c,1}$	9.0			-
Compliance time constant	$t_{c,1}$	5.0			min
Systemic resistance scaling constant	$m_{sys,1}$				
Systemic resistance scaling constant	$m_{sys,2}$				
Systemic resistance time constant	$t_{sys,1}$				
Systemic resistance time constant	$t_{sys,2}$				

References

1. Coceani F, Baragatti B. Mechanisms for Ductus Arteriosus Closure. Seminars in Perinatology 2012;36:92–7.

2. Munneke AG, Lumens J, Delhaas T. Cardiovascular fetal-to-neonatal transition: an in silico model. Pediatr Res 2022;91:116–28.

3. Walther FJ, Benders MJ, Leighton JO. Early changes in the neonatal circulatory transition. The Journal of Pediatrics 1993;123:625–32.

4. Vonderen JJ van, Pas AB te, Kolster-Bijdevaate C, et al. Non-invasive measurements of ductus arteriosus flow directly after birth. Archives of Disease in Childhood - Fetal and Neonatal Edition 2014;99:F408–12.

5. Maeno YV, Kamenir SA, Sinclair B, van der Velde ME, Smallhorn JF, Hornberger LK. Prenatal Features of Ductus Arteriosus Constriction and Restrictive Foramen Ovale in d-Transposition of the Great Arteries. Circulation 1999;99:1209–14.

6. Lachaud M, Dionne A, Brassard M, et al. Cardiac hemodynamics in fetuses with transposition of the great arteries and intact ventricular septum from diagnosis to end of pregnancy: longitudinal follow-up. Ultrasound in Obstetrics & Gynecology 2021;57:273–81.

7. Schmölzer GM, Hooper SB, Wong C, Kamlin COF, Davis PG. Exhaled Carbon Dioxide in Healthy Term Infants Immediately after Birth. The Journal of Pediatrics 2015;166:844-849.e3.

8. Palme-Kilander C, Tunell R, Chiwei Y. Pulmonary gas exchange immediately after birth in spontaneously breathing infants. Arch Dis Child 1993;68:6–10.

9. Dawes GS, Mott JC, Widdicombe JG, Wyatt DG. Changes in the lungs of the new-born lamb. J Physiol 1953;121:141–62.

10. Yigit MB, Kowalski WJ, Hutchon DJR, Pekkan K. Transition from fetal to neonatal circulation: Modeling the effect of umbilical cord clamping. Journal of Biomechanics 2015;48:1662–70.

11. Alsaied T, Tseng S, King E, et al. Effect of fetal hemodynamics on growth in fetuses with single ventricle or transposition of the great arteries. Ultrasound in Obstetrics & Gynecology 2018;52:479–87.

12. Charbonneau L, Chowdhury RA, Marandyuk B, et al. Fetal cardiac and neonatal cerebral hemodynamics and oxygen metabolism in transposition of the great arteries. Ultrasound in Obstetrics & Gynecology 2023;61:346–55.

13. Hooper SB, Polglase GR, Roehr CC. Cardiopulmonary changes with aeration of the newborn lung. Paediatr Respir Rev 2015;16:147–50.

14. Sobotka KS, Hooper SB, Allison BJ, et al. An Initial Sustained Inflation Improves the Respiratory and Cardiovascular Transition at Birth in Preterm Lambs. Pediatr Res 2011;70:56–60.

15. Sá-Couto CD, Andriessen P, Van Meurs WL, Ayres-De-Campos D, Sá-Couto PM. A Model for Educational Simulation of Hemodynamic Transitions at Birth. Pediatric Research 2010;67:158–65.

16. Coskun S, Yüksel H, Bilgi Y, Lacin S, Tansug N, Onag A. Non-invasive evaluation of the adaptations of cardiac function in the neonatal period: a comparison of healthy infants delivered by vaginal route and caesarean section. Acta Med Okayama 2001;55:213–8.

17. Kishkurno S, Takahashi Y, Harada K, Ishida A, Tamura M, Takada G. Postnatal Changes in Left Ventricular Volume and Contractility in Healthy Term Infants. Pediatr Cardiol 1997;18:91–5.

18. Shiota T, Harada K, Takada G. Left ventricular systolic and diastolic function during early neonatal period using transthoracic echocardiography. Tohoku J Exp Med 2002;197:151–8.

19. Doni D, Nucera S, Rigotti C, et al. Evaluation of hemodynamics in healthy term neonates using ultrasonic cardiac output monitor. Ital J Pediatr 2020;46:112.

20. Hillman NH. Increasing pulmonary blood flow at birth: the nerve of the baby. J Physiol 2017;595:1437–8.

21. Mynard JP. Computer modelling and wave intensity analysis of perinatal cardiovascular function and dysfunction. 2011;
